# Evaluation of Physiological Effects Induced by Manuka Honey Upon *Staphylococcus aureus* and *Escherichia coli*

**DOI:** 10.3390/microorganisms7080258

**Published:** 2019-08-13

**Authors:** Patricia Combarros-Fuertes, Leticia M. Estevinho, Rita Teixeira-Santos, Acácio G. Rodrigues, Cidália Pina-Vaz, Jose M. Fresno, M. Eugenia Tornadijo

**Affiliations:** 1 Department of Food Hygiene and Technology, Faculty of Veterinary Science, University of León, Campus de Vegazana, 24071 León, Spain; 2 Mountain Research Center (CIMO), Polytechnic Institute of Bragança, Campus Santa Apolónia, 5301-855 Bragança, Portugal; 3 Division of Microbiology, Department of Pathology, Faculty of Medicine, University of Porto, 4200-319 Porto, Portugal; 4 Center for Research in Health Technologies and Information Systems (CINTESIS), Faculty of Medicine, University of Porto, 4200-450 Porto, Portugal; 5 Burn Unit, Department of Plastic and Reconstructive Surgery, Hospital São João, 4200-319 Porto, Portugal

**Keywords:** manuka honey, flow cytometry, antibacterial effects, bacteria models, metabolic activity, efflux pump

## Abstract

Several studies have explored the antimicrobial properties of manuka honey (MkH). However, the data available regarding antibacterial action mechanisms are scarcer. The aim of this study was to scrutinize and characterize primary effects of manuka honey (MkH) upon the physiological status of *Staphylococcus aureus* and *Escherichia coli* (as Gram-positive and Gram-negative bacteria models, respectively), using flow cytometry (FC) to reveal its antibacterial action mechanisms. Effects of MkH on membrane potential, membrane integrity and metabolic activity were assessed using different fluorochromes in a 180 min time course assay. Time-kill experiments were carried out under the same conditions. Additionally, MkH effect on efflux pumps was also studied in an *E. coli* strain with an over-expression of several efflux pumps. Exposure of bacteria to MkH resulted in physiological changes related to membrane potential and membrane integrity; these effects displayed slight differences among bacteria. MkH induced a remarkable metabolic disruption as primary physiological effect upon *S. aureus* and was able to block efflux pump activity in a dose-dependent fashion in the *E. coli* strain.

## 1. Introduction

The emergence of multi-drug-resistant pathogens is one of the most pressing concerns of public health and food industry worldwide; the situation is especially disturbing in the case of bacteria [1,2].

The excessive and inadequate use of antibiotics clearly contributes to the development of resistance, usually through genetic changes [3]. Community and hospital settings represent the principal ecological niches of resistance emergence in human health. Moreover, the intensification of food animal production, led to an extensive antibiotics administration, not only as therapeutics, but also as prophylactics or growth promoters increasing the emergence and spread of drug-resistance [2,4]. As a consequence, some common infections have become extremely difficult, or even impossible, to treat [1,5]. Indeed, the World Health Organization predicts that infections involving antibiotic resistant pathogens will pose major patient care management issues, which inevitably increases in the hospital stays, patient morbidity and mortality [6]

*Staphylococcus aureus* and *Escherichia coli* are commonly involved in infections of both community and healthcare settings and are amongst the leading causative agents of food-borne infections worldwide. In addition, according to the last surveillance of antimicrobial resistance in Europe, these bacterial species currently present resistance to multiple antimicrobial groups [3]. The above scenario stresses the need to uncover new therapeutic or co-adjuvant options, ideally not prone to resistance development. In this context, several studies have explored the antibacterial properties and mechanisms of action of manuka honey (MkH) [7,8,9] which is produced from *Leptospermum* species, native to New Zealand and Australia. The antimicrobial activity of this variety of honey is connected with common factors such as, osmotic pressure (as result of the high concentration of sugars), acidity or oxidative stress. Furthermore, MkH exhibits non-peroxide antibacterial activity related to the presence, in high concentrations, of compounds such as methylgloxal or leptosin [10,11].

Despite the numerous studies on the antibacterial properties of honey it use as an antibacterial agent remains underestimated, partly due to the lack of comprehensive scientific evidence supporting its use [9,12,13]. Therefore, more investigations elucidating the role that MkH could play as an antimicrobial agent are necessary.

Flow cytometry (FC) has been widely applied to study the antimicrobial susceptibility and characterization of resistance mechanisms of several microorganisms [14,15,16,17,18]. Unlike other techniques, FC goes beyond classical methodologies in which bacterial viability is generally equated with the ability to grow and subsequently generate a colony of cells. However, for some bacterial cells the viability is not so strictly related to grow capacity. Indeed, although bacteria may lack the ability to reproduce and grow under certain conditions, cells may still possess many of the properties of the fully functional viable cells [19,20]. In addition, using specific fluorochromes, FC allows to evaluate the physiological states of bacteria, which confirm the phenotypic changes suggested by other methodologies, such as genomics or proteomics [19].

The aim of this study was to evaluate the physiological changes induced by MkH upon *Escherichia coli* and *Staphylococcus aureus* in a time course assay, using a FC protocol which was adapted for the purpose. This research is essential to understand honey’s targets when acting as an antimicrobial and to further explore the therapeutic potential of this natural product.

## 2. Results

### 2.1. Manuka Honey-Induced Alterations in S. aureus CECT 86 and E. coli CECT 515

#### 2.1.1. Effects of MkH on Cell Viability

**Figure 1:**
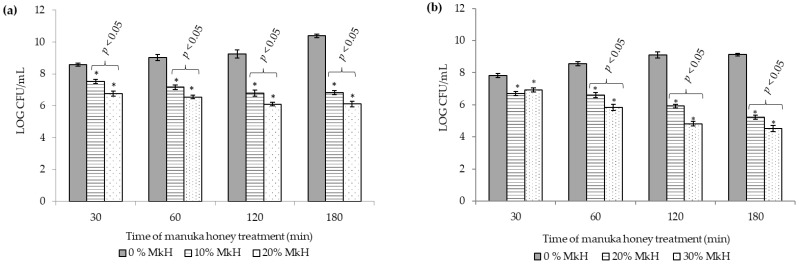
Effect of MkH on *S. aureus* CECT 86 (**a**) and *E. coli* CECT 515 (**b**) cell viability assessed by colony forming units (CFU) counting (expressed in Log_10_ units) by plating on Mueller–Hinton Agar. Data at each time point corresponds to mean ± standard deviation. NS indicate no significant differences and asterisks indicate significant differences (*: *p* < 0.05; **: *p* < 0.01; ***: *p* < 0.001) between treated cells vs. the control (non-treated cells). *p*-values, indicate significant differences (*p* < 0.05) between the two honey concentrations tested.

Figure 1 represents reference strains viability (*S. aureus* CECT 86 (a) and *E. coli* CECT 515 (b)) following application of the two MkH concentrations under test in comparison to the non-treated cells.

A significant growth reduction was registered soon after 30 min of incubation with both concentrations (*p* < 0.05). CFU counts of the treated *S. aureus* decreased progressively throughout time up to 120 min of treatment, time in which CFU counts stabilized (Figure 1a). On the other hand, *E. coli* viability decreased progressively along the time up to the end of the treatment (*p* < 0.05) (Figure 1b). Significant differences between honey-treated and non-treated cells, and between both treatment concentrations tested on each microorganism were observed at all time points sampled (*p* < 0.05).

#### 2.1.2. Effects of MkH on Cytoplasmic Membrane Potential

**Figure 2:**
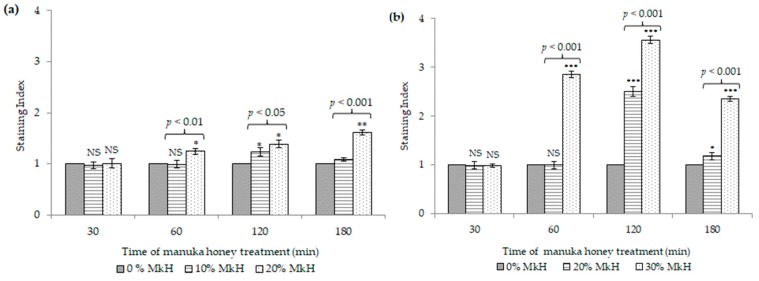
Effect of MkH over membrane potential of *S. aureus* CECT 86 (**a**) and *E. coli* CECT 515 (**b**), evaluated using DiBAC4 (3) staining. Data at each time point corresponds to mean ± standard deviation. NS indicate no significant differences and asterisks indicate significant differences (*: *p* < 0.05; **: *p* < 0.01; ***: *p* < 0.001) between treated cells vs. the control (non-treated cells). *p*-values, indicate significant differences (*p* < 0.05) between the two honey concentrations tested.

Figure 2 represents the effects of MkH on the cytoplasmic membrane potential of *S. aureus* CECT 86 (a) and *E. coli* CECT 515 (b), in comparison to non-treated cells.

*S. aureus* treated with 10% (*w*/*v*) of MkH displayed a significant increase of fluorescence after 120 min of incubation (*p* < 0.05). Interestingly, after this time, cell membrane seemed to repolarize, re-establishing their membrane potential (SI = 1.08; *p* = 0.068) (Figure 2a). Conversely, treatment with 20% (*w*/*v*) of MkH resulted in a time-dependent increase of depolarized cells from 60 min (SI = 1.24; *p* < 0.05) up to 180 min (SI = 1.61; *p* < 0.01) of treatment. Significant differences were observed between both treatments at 60 min (*p* < 0.01), 120 min (*p* < 0.05) and 180 min (*p* < 0.001).

Regarding *E. coli*, cells treated with 20% (*w*/*v*) of MkH displayed a significant increase of fluorescence after 120 min of incubation (SI = 2.50; *p* < 0.001) (Figure 2b). The application of 30% (*w*/*v*) of MkH resulted in a time-dependent increase in the amount of depolarized cells between 60 min (SI = 2.85; *p* < 0.001) and 120 min (SI = 3.56; *p* < 0.001) of exposition (Figure 2b). Interestingly, after 120 min, cells seemed to repolarize progressively under both treatment conditions. Significant differences were observed between the two treatments at 60 min (*p* < 0.001), 120 min (*p* < 0.001) and 180 min (*p* < 0.001).

*E. coli* cells exhibited higher membrane depolarization than *S. aureus* cells exposed to the same concentration of MkH (20% (*w*/*v*)). However, *E. coli* cells seemed to be able to restore MP progressively after 120 min of treatment, regardless of the honey concentration.

These findings support the relevance of honey concentration and time of exposition upon the increase of cell membrane depolarization in both bacteria.

#### 2.1.3. Effects of MkH on Membrane Integrity

**Figure 3:**
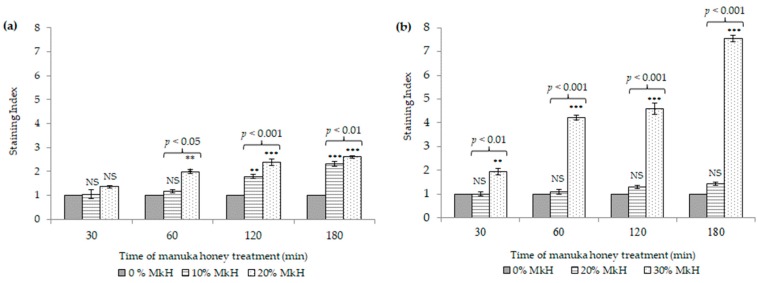
Effect of MkH over membrane integrity of *S. aureus* CECT 86 (**a**) and *E. coli* CECT 515 (**b**), evaluated using propidium iodide staining. Data at each time point corresponds to mean ± standard deviation. NS indicate no significant differences and asterisks indicate significant differences (*: *p* < 0.05; **: *p* < 0.01; ***: *p* < 0.001) between treated cells vs. the control (non-treated cells). *p*-values, indicate significant differences (*p* < 0.05) between the two honey concentrations tested.

Figure 3 represents the effects of MkH on the membrane integrity of *S. aureus* CECT 86 (a) and *E. coli* CECT 515 (b) compared with non-treated cells.

After MkH exposure, minor—yet significant—modifications in *S. aureus* membrane were observed (Figure 3a), but not as an initial event. Indeed, significant differences in the SI were observed in cells treated with 10% of MkH after 120 min of treatment (SI = 1.79; *p* < 0.01) increasing up to 180 min (SI = 2.31; *p* < 0.001). On the other hand, cells treated with 20% of MkH showed PI-intake after 60 min (SI = 2.00; *p* < 0.001), increasing progressively following 120 and 180 min of treatment (SI = 2.38; *p* < 0.001 and SI= 2.61; *p* < 0.001, respectively). Significant differences were also registered between both experimental conditions at 60 min (*p* < 0.05), 120 min (*p* < 0.001) and 180 min (*p* < 0.01).

Differently, *E. coli* treated with 20% of MkH showed no significant differences when compared with viable non-treated cells. Conversely, the treatment of *E. coli* cells with 30% (*w*/*v*) of MkH resulted in a time-dependent increase of SI from the early stages of the treatment at 30 min (SI = 1.96; *p* < 0.01) up to the end at 180 min (SI = 7.54; *p* < 0.001). Significant differences were observed between the two treatments conditions at all time points.

#### 2.1.4. Effects of MkH on Metabolic Activity

**Figure 4:**
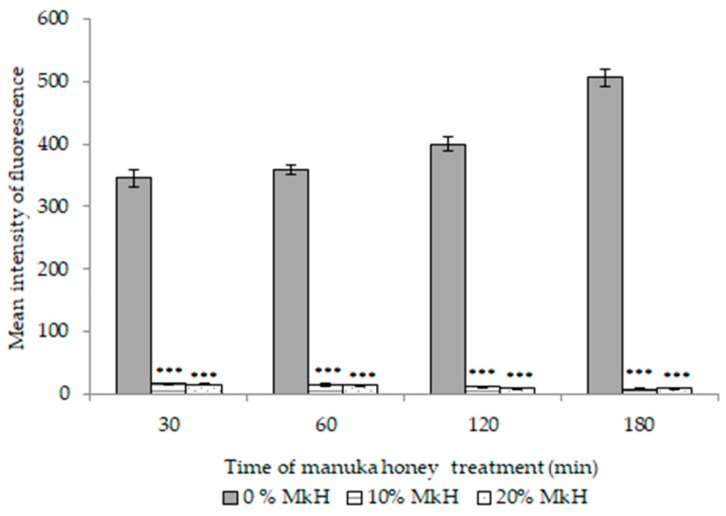
Effect of MkH on *S. aureus* CECT 86 metabolic activity, evaluated using calcein-AM staining. Data at each time point corresponds to mean ± standard deviation. NS indicate no significant differences and asterisks indicate significant differences (*: *p* < 0.05; **: *p* < 0.01; ***: *p* < 0.001) between treated cells vs. the control (non-treated cells). *p*-values, indicate significant differences (*p* < 0.05) between the two honey concentrations tested.

Figure 4 depicts the metabolic effects of MkH on *S. aureus* CECT 86 cells, presented as MIF (mean intensity of fluorescence values).

MIF displayed by viable non-treated cells increased slightly up for 180 min but without significant differences along the time. Soon after 30 min of treatment with 10% (*w*/*v*) of MkH MIF decreased to a value of 15.87 (*p* < 0.001) and decreased progressively up to the end of treatment (MIF = 7.33; *p* < 0.001). The drastic reduction observed on MIF values could be explained by an extremely reduced metabolic activity or by the loss of membrane integrity, which may lead to the loss of fluorescence. Cells treated with 10% (*w*/*v*) of MkH showed significant PI-intake after 120 min of treatment. However, metabolic activity decreased after 30 min of exposure at a similar concentration of honey. Thus, the second hypothesis may be discarded, at least during the first stages of the treatment.

Although slight differences were registered between both treatment conditions, they were not statistically significant (Figure 4). In addition, no significant differences were observed between MkH-treated cells and death control (data not shown), that is cells after 120 min of treatment at 10% (*w*/*v*) of MkH (MIF = 8.56; *p* = 0.324) and 60 min of treatment at 20% (*w*/*v*) (MIF = 15.08; *p* = 0.055). This data suggests that metabolic inactivation is less dependent from MkH concentration and treatment time than membrane depolarization and membrane lesion.

### 2.2. Manuka Honey Effect on Efflux Pumps in E. coli AG100_TET_

**Figure 5:**
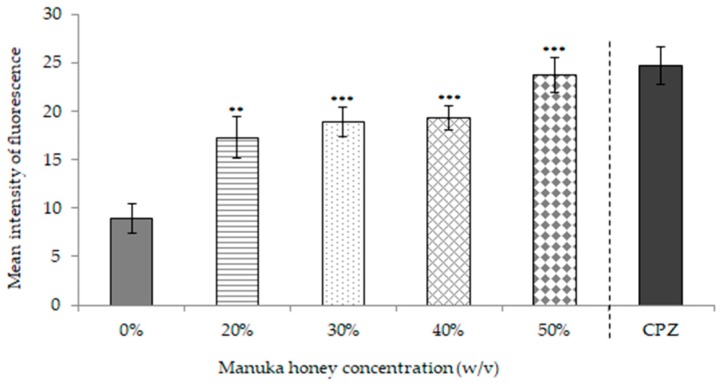
Effect of MkH on *E. coli* AG100_TET_ efflux pump activity evaluated using ethidium bromide staining. Chlorpromazine (CPZ) was used as efflux pump inhibitor.

The results obtained regarding efflux pumps blockade of *E. coli* AG100_TET_ are presented in Figure 5. Viable non-treated bacteria were able to expulse EtBr so that the MIF was lower (MIF = 8.95) compared to honey treated bacteria and to positive control (bacteria treated with chlorpromazine (CPZ)). MIF of cells exposed to honey increased significantly at all concentrations tested 20% (*w*/*v*) (MIF = 17.32; *p* < 0.01), 30% (*w*/*v*) (MIF = 18.92; *p* < 0.001), 40% (*w*/*v*) (MIF = 19.28; *p* < 0.001) and 50% (*w*/*v*) (MIF = 23.73; *p* < 0.001). In addition, no significant differences were observed between MkH-treated cells at 50% (*w*/*v*) and positive control cells (MIF = 24.72; *p* = 0.475), suggesting that MkH promotes efflux pumps blockade in a dose-dependent fashion.

## 3. Discussion

The emergence of multi-drug-resistant bacteria poses a great worldwide problem for the agriculture and food industries and rises public health concerns [1,2]. This situation makes necessary to search for new effective alternatives. Antibacterial activity of MkH has been described in numerous studies, which demonstrate that this type of honey could be promising as adjuvant therapy [8]. However, more studies to elucidate how honey acts against bacteria are necessary [11,21,22,23].

The present study provides new insights regarding the physiological effects of MkH upon *S. aureus* and *E. coli* through FC. The ability of bacteria to deal with environmental stresses determines their success to survive. Following the exposure to MkH, several physiological changes were registered, both in *S. aureus* and *E. coli*. The effects of MkH on cell viability, cytoplasmic membrane potential and membrane integrity were evaluated on *S. aureus* CECT 86 and *E. coli* CECT 515. Effects of MkH on metabolic activity were assessed on *S. aureus* CECT 86. Finally, the effect of MkH on efflux pump activity was valued in *E. coli* AG100_TET_ strain.

A significant reduction of bacterial growth was observed after MkH exposure. This data suggest that *S. aureus* and *E. coli* cells were not able to recover from the MkH induced alterations and lost their viability as result of physiological changes. This effect seems to be irreversible up to the maximum time tested, corroborating data published in other studies [9,12].

An essential aspect of bacterial adaptation to environmental conditions is the equilibrium of ion concentration inside and outside of bacteria, which determines cell membrane potential (MP) [22]. In this study, bacterial MP alteration was confirmed by DiBAC4 (3) staining, although it was not a relatively early event in both bacteria (Figure 2). However, under some treatment conditions, reversible effects and repolarization of cell membrane were observed, which evidence that other underlying mechanisms that caused bacterial death happened. Indeed, previous studies, using transcriptomics and proteomics, described alterations in the expression level of some stress response genes [23,24]. Nevertheless, the physiological changes on MP of *S. aureus* and *E. coli* were not previously described.

On the other hand, the loss of membrane integrity represents a meaningful damage for bacteria. The results obtained in this study reveal that the treatment time and specially the MkH concentration are key factors regarding the induction of membrane injury in both bacteria. Moreover, these results evinced that the effect of MkH on membrane integrity seems to be slightly different between Gram-positive and Gram-negative bacteria. Previous studies have also detected such differences among both types of bacteria, while limited or no evidence of cell lysis of *S. aureus* after MkH treatment was found [12,25]. In contrast, for *Pseudomonas aeruginosa* (another Gram-negative bacteria), there was evidence that large amounts of extracellular material result of cellular lysis [26,27]. In addition, a reduction in *oprF* gene expression in *P. aeruginosa* population treated with MkH was related to deficiencies on membrane integrity [27]. For *E. coli*, such deficiencies might be related to *ompA* gene, orthologue to *oprF* with significant amino acid similarity [28]. Nevertheless, genomic studies are necessary to confirm this assumption.

The influence of MkH on *S. aureus* metabolism was studied using calcein-AM. This compound is a cell-permeant esterase substrate that measure enzymatic activity [20]. Esterases encompass a large and sundry group of enzymes able to catalyse the cleavage and formation of carboxyl ester bonds. Taking into account the results obtained, MkH induced a significant metabolism disruption on *S. aureus* cells as an early event. Previous studies, comprising genomics and/or proteomics, demonstrated that MkH induces a reduction in the expression of genes and proteins involved in energy metabolism of *S. aureus* [9,10]. However, it has been described that changes in gene expression does not necessarily concur with a bacterial physiological response to stress or with the altered levels of protein expression determined by proteomic analysis [9]. The considerable repercussion upon metabolic bacterial physiology observed in this study might be related to the previously described genomic alterations. The effect of MkH on metabolic activity of *E. coli* cells was not assessed because Gram-negative bacteria are not permeable to calcein-AM [29]. Nevertheless, the results obtained using *S. aureus* encourage the search of other alternatives to evaluate metabolic disruption. Moreover, studies on *E. coli* [23], as well as on *P. aeruginosa* [30] confirmed the effect of MkH on the ability of bacteria to scavenge iron, which is essential for bacterial metabolism and survival. The assessment of MkH influence on metabolic activity of *E. coli* using FC, could confirm metabolic status alterations, which might be related with the results observed in other studies.

The effect of MkH on efflux pump activity was also assessed on *E. coli* AG100_TET_, a bacterial strain which over-expresses several efflux pumps when exposed to high concentrations of tetracycline. Efflux pumps can be specific to antibiotics, although most of them are multidrug transporters that are capable to pump a wide range of unrelated compounds and thus, significantly contribute to multidrug resistance [31,32]. Some studies have demonstrated the synergism or additive antibacterial effects between MkH and some antibiotics such as tetracycline, rifampicin, imipinem or mupirocin [13,33,34,35]. In spite of that, the underlying mechanisms were not suggested. These findings strongly suggest that MkH is able to block efflux activity. Conversely, a previous study addressing the global action of MkH on *E. coli* demonstrated that following exposure, some genes belonging to the *EvgAS* regulon—used by *E. coli* in adaptive responses to acid, osmotic and drug resistance—were upregulated [23]. This disparity could indicate no correlation between gene expression and bacterial physiological response or might be related with a possible metabolic disruption, since antibiotic efflux is an energy-dependent mechanism. To validate this last hypothesis, it would be necessary to confirm this specific MkH effect on *E. coli*.

This study demonstrated that MkH induced effects on the physiology of bacteria, as result of their effort to adapt and overcome from a stressful environment. MkH effects did not occur as an initial event, with the exception of metabolic disruption, and were dependent on honey concentration. However, the final result observed was the reduction of cell viability. Due to its complex composition, with multiple active components, MkH acts in a multifactorial way upon several cellular target sites, as was confirmed in this study, which hinders the development of bacterial specific defence mechanisms and, therefore, resistance [13,36].

## 4. Conclusions

The global escalation in antibiotic resistance means that alternative antimicrobials are essential. Our results demonstrate that MkH induced a remarkable metabolic disruption as primary physiological effect on *S. aureus*. Other effects, such as the membrane potential imbalance and the membrane injury were also detected in both reference strains. The efflux pump blockade observed in *E. coli* AG100_TET_ was dependent on honey concentration. This finding could encourage further studies to know the effect of MkH-antibiotic combinations over bacterial physiological effect, addressing especially those which are usually extruded from bacteria by efflux mechanisms.

Data presented in this study are in accordance with previous findings on antibacterial action mechanisms of MkH, carried out using other methodologies. This fact corroborate that FC is a valuable tool for analysing physiological states of bacteria exposed to honey.

Further studies using other varieties of honey with demonstrated antibacterial activity are necessary to elucidate whether the action mechanisms described for MkH are similar in other types of honey.

## 5. Materials and Methods

### 5.1. Bacterial Strains, Growth Conditions and Inoculated Broth Preparation

*Staphylococcus aureus* (CECT 86) and *Escherichia coli* (CECT 515) from the Spanish Type Culture Collection were used as Gram-positive and Gram-negative bacteria models, respectively. Reference strains were used instead of clinical or resistant isolates in order to avoid a possible altered response mechanism to MkH exposure, which could confound the effects of honey upon bacteria. In addition, *E. coli* AG100_TET_ (kindly provided by Dr. Miguel Viveiros), an AG100 progeny strain, which over-expresses several efflux pumps when exposed to high concentrations of tetracycline [37], was used for studying MkH effect on efflux pumps. Prior to experiments, bacteria were subcultured twice in MH agar to ensure the purity of cultures.

**Figure 6:**
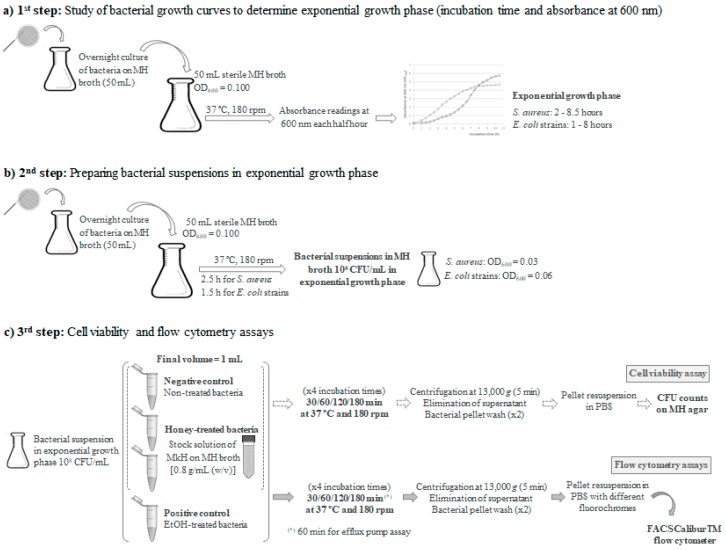
Flow cytometry protocol to evaluate the antibacterial mechanisms of manuka honey.

In order to analyse the kinetics of bacterial growth and to determine, the bacteria exponential phase, a study of the growth curves was carried out. For this purpose, 100 mL of Mueller Hinton (MH; Sigma-Aldrich, St. Louis, Mo., USA) broth were inoculated in an Erlenmeyer flask with an overnight culture of bacteria to obtain an optical density OD_600_ absorbance of 0.100, approximately corresponding to 1 × 10^8^ CFU/mL. This culture was prepared by transferring an isolated colony (from a fresh culture in Mueller Hinton (MH) agar) into MH broth; overnight culture was then incubated for 16 h in 50 mL of MH broth. The flask was placed in a shaker incubator at 37 °C and 180 rpm. Aliquots were taken every half hour to read the absorbance at 600 nm until growth curves reached stationary phase. At the described conditions, the exponential growth phase of *S. aureus* occurred between 2 h and 8.5 h, and between 1 h and 8 h for *E. coli* strains (Figure 6a).

All flow cytometric assays were performed using a cell suspension prepared with MH broth and bacteria in initial exponential growth phase. Briefly, following the same conditions used for the study of growth curves, sterile MH broth was inoculated with an overnight culture of bacteria until reaching OD_600_ absorbance of 0.100. Once the exponential phase was reached after incubation at 37 °C and 180 rpm during 2.5 h for *S. aureus* and 1.5 h for *E. coli* strains, a bacteria cell suspension corresponding to 10^8^ bacteria cells/mL was prepared for the flow cytometric assays (Figure 6b).

### 5.2. Bacterial Susceptibility to Manuka Honey

Manuka honey MGO 550+ (containing at least 550 mg of methylglyoxal per kg of honey) (Manuka Health, New Zealand) was used in this study. Minimal inhibitory concentration (MIC) and minimal lethal concentration (MLC) were previously determined according to the Clinical and Laboratory Standards Institute M07-A9 protocol [38]. MIC and MLC were defined as 10% (*w*/*v*) for *S. aureus* and 20 % (*w*/*v*) for *E. coli* strains [39].

A stock solution of 0.8 g/mL of the MkH sample (the maximum concentration of honey able to solubilize) in MH sterile broth was prepared in sterile Falcon tubes to carry out the following assays. Complete dissolution of honey was achieved with intense agitation.

### 5.3. Evaluation of Cell Viability

The evaluation of cell viability was performed for *S. aureus* CECT 86 and *E. coli* CECT 515. The concentrations of MkH tested were selected according to the susceptibility results: 10% and 20% (*w*/*v*) for *S. aureus* and 20% and 30% (*w*/*v*) for *E. coli* [39]. The final volume was 1 mL in all assays. In sterile Eppendorf tubes, variable volumes (between 0.625 and 0.875 mL) of the inoculums (10^8^ bacteria cells/mL in exponential growth phase) and variable volumes (between 0.125 and 0.375 mL) of the stock solution of MkH to achieve the target concentrations were mixed and incubated for 30, 60, 120 and 180 min at 37 °C and 180 rpm (Figure 6c).

After each treatment, bacterial suspensions were centrifuged at 13,000× *g* (Eppendorf Centrifuge 5804R, Hamburg, Germany) for 5 min. The supernatant was discarded and the pellets were washed twice with 1 mL of phosphate saline buffer (PBS; Sigma-Aldrich, St. Louis, Mo., USA) in order to prevent antibacterial carryover. Afterwards, bacteria were resuspended with 1 mL of PBS. Cell viability was determined by counting colony forming units per mL (CFU/mL) (Figure 6c). Briefly, the number of viable cells for each treatment was determined by plating 100 μL of serial dilutions on MH agar plates. The number of CFU/mL was determined and compared with that of the control plates (not exposed to honey) after incubation at 37 °C for 24 h.

### 5.4. Functional Characterization of Manuka Honey-Induced Action

The physiological status of bacterial cells following exposure to different concentrations of MkH in a time course assay were assessed by FC. Previous FC assays [14,21,40,41] were adapted to honey peculiarities. For protocol optimisation, several important factors were taken into account, among which the microorganisms involved, the fluorochromes and its concentration, honey concentrations and incubation times [18].

Following the same protocol used in the evaluation of cell viability, the appropriate volumes of the inoculated MH broth and a MkH dilution in sterile broth to achieve the target concentrations of MkH ((10% and 20% (*w*/*v*) for *S. aureus* CECT 86, 20% and 30% (*w*/*v*) for *E. coli* CECT 515) were incubated in sterile Eppendorf tubes at 37°C and 180 rpm for different times (30, 60, 120 and 180 min). In all assays, the final volume was 1 mL. For each strain, non-treated cells were used as negative control and cells treated with 1 mL of ethanol 70% (*w*/*w*) as positive control (Figure 6c).

After each treatment, the pellets were obtained and washed twice with 1 mL of PBS and then resuspended and stained with 1 mL of PBS containing the different fluorochromes at the concentrations detailed below. In addition, non-treated and non-stained cells were also analysed in order to evaluate the native cell autofluorescence.

All cytometric assays were performed using a standard FACS Calibur TM (BD Biosciences, Franklin Lakes, NH, USA) equipped with 3 photomultipliers, standard filters, and a 15 mW, 488 nm argon laser, using Cell Quest Pro software version4.0.2 (BD Biosciences, Franklin Lakes, NJ, USA). Data was recorded for 20,000 cells for each assay.

Due to honey characteristics and the sensitivity of some fluorochromes to acidity, the pH evaluation was performed for each assay.

#### 5.4.1. Assessment of Membrane Potential

The effect of MkH on cell membrane potential of the reference strains was evaluated using bis-(1,3-dibutylbarbituric acid) trimethineoxonol (DiBAC4 (3); Sigma-Aldrich, Munich, Germany) as described by Teixeira-Santos et al. (2015). After honey treatment, cells were incubated for 15 min in the dark at 37 °C and 180 rpm with 0.5 mg/L of DiBAC4 (3). DiBAC4 (3) enters only depolarized cells, where it binds reversibly to membrane and intracellular components, resulting in an increased fluorescent signal [20]. The fluorescence intensity (FI) was registered at FL1 (absorbance at 530 nm). A staining index (SI) was defined as the ratio between the FI of treated cells and the FI of non-treated cells.

#### 5.4.2. Assessment of Membrane Integrity

Cell membrane integrity of the two reference strains was assessed with propidium iodide (PI; Sigma-Aldrich, Munich, Germany) staining. After honey treatment, bacteria were stained with 1 mg/L of PI for 30 min at 37 °C and 180 rpm in darkness. PI is a cell viability marker which enters cells only when membrane has been seriously injured and exhibits fluorescence after binding to nucleic acids [20]. FI was measured at FL3 (Absorbance at 630 nm). A SI was also defined, as the ratio between the FI of treated cells and the FI of non-treated cells.

#### 5.4.3. Assessment of Metabolic Activity

Metabolic changes induced by MkH on *S. aureus* CECT86 were evaluated using the fluorogenic substrate calcein-AM (Sigma-Aldrich, Munich, Germany) at a concentration of 1 mg/L for 120 min at 37 °C and 180 rpm in darkness. Only metabolically active cells are able to hydrolyse calcein-AM to a fluorescent compound by intracellular esterases. This compound accumulates inside cells with intact membranes and increases their fluorescence. When cells have an injured membrane, impermeant end products will be quickly lost to the extracellular space, resulting to a decreased fluorescent signal [20]. FI was registered at FL1. Mean intensity of fluorescence (MIF) was used to evaluate treatment differences.

#### 5.4.4. Assessment of Efflux Pumps

The effect of MkH on efflux pumps was evaluated in *E. coli* AG100_TET_ strain, following the protocol described by Paixão et al. (2009) involving accumulation and efflux assays using 1 mg/L of ethidium bromide (EtBr; Sigma-Aldrich, Munich, Germany). Bacteria treated with 20 mg/L of chlorpromazine (CPZ; Sigma-Aldrich Química SA Madrid, Spain), an efflux pump inhibitor, was used as positive control. EtBr is a common efflux pump substrate; under normal conditions EtBr enters the bacteria and is rejected to outside by efflux pump action. In case these pumps are blocked EtBr accumulates inside the bacteria, increasing its fluorescence [37].

In this assay four concentrations of MkH (20%, 30%, 40% and 50%) were tested. Bacterial suspensions were incubated for 60 min at 37 °C and 180 rpm in darkness. The fluorescence intensity (FI) was measured at FL3. MIF was used to evaluate treatment differences.

### 5.5. Statistical Analysis

All experimental procedures were replicated three times using fresh culture preparations; the results were expressed in all cases as mean values and respective standard deviations

The statistical analysis was performed using IBM SPSS statistics v.24.0 (SPSS, Chicago, IL, USA). All variables were tested for the assumptions of normality and homoscedasticity. Paired-sample Student’s t-test or Wilcoxon signed rank test (parametric or nor parametric test depending on the characteristics of the variables) were used to evaluate if there were any differences between non-treated bacteria and MkH treated bacteria at the different time points sampled in each assay. In addition, unpaired-sample Student’s t-test or Mann-Whitney test (parametric or nor parametric test depending on the characteristics of the variables) were used to compare the differences between the two MkH concentrations tested in each assay. *p* < 0.05 was considered to be significant.
